# A Dual-Signal Electrochemiluminescence Sensor for Kanamycin Detection Based on a Self-Enhanced Zr MOF and Single Co-Reactant Competition Mechanism

**DOI:** 10.3390/bios15050291

**Published:** 2025-05-05

**Authors:** Yawen Zhu, Xuemei Wang, Zhiyong Yan, Feifei Zhang, Jianfei Xia, Lili Lv, Zonghua Wang

**Affiliations:** 1 Instrumental Analysis Center of Qingdao University, College of Chemistry and Chemical Engineering, Qingdao Application Technology Innovation Center of Photoelectric Biosensing for Clinical Diagnosis and Treatment, Qingdao University, Qingdao 266071, China; zhuyawen@qdu.edu.cn (Y.Z.); 2021010016@qdu.edu.cn (X.W.); zhangfeifei@qdu.edu.cn (F.Z.); xiajianfei@qdu.edu.cn (J.X.); 2 Institute of Analysis and Testing, Beijing Academy of Science and Technology, Beijing Center for Physical and Chemical Analysis, Beijing 100089, China; yanzhiyong@bcpca.ac.cn; 3 Characteristic Laboratory of Advanced Metal Functional Materials and Processing in Universities of Shandong, School of Mechanical and Electronic Engineering, Qingdao Binhai University, Qingdao 266555, China

**Keywords:** self-enhanced electrochemiluminescence, co-reactant competition, dual-signal, zirconium metal organic framework, luminol, kanamycin

## Abstract

The dual-signal output self-calibration mode reduces the false positive and negative signals of electrochemiluminescence (ECL) aptamer sensors. A competitive dual-signal ECL platform was designed for the ultrasensitive detection of kanamycin (KAN) using a zirconium metal–organic framework (Zr MOF) and Luminol as ECL emitters. To enhance the ECL efficiency, a co-reactant (polyethyleneimine, PEI) was covalently bound to the Zr MOF to achieve self-enhanced ECL. Based on the selective interaction between KAN and its aptamer, the Luminol/KAN/Zr MOF-PEI “sandwich” structure was immobilized on the electrode surface. The competition for PEI between emitters increased the Luminol ECL signal and decreased the Zr MOF’s ECL signal. The ratio in ECL signals between the two competitive emitters enabled the quantitative analysis of KAN, achieving a detection limit as low as 7.86 × 10^−4^ ng/mL. This study elucidated the synergistic mechanism between self-enhanced ECL and ECL competition, offering a novel approach for constructing dual-signal ECL sensors using a single co-reactant.

## 1. Introduction

Kanamycin (KAN), a powerful inhibitor of protein synthesis, has been extensively used in medicine and as an additive in livestock feed [1,2]. However, the excessive use of KAN has resulted in significant accumulation in humans, aquatic environments, animals, and plants, thereby posing substantial challenges to health and environmental safety [3,4,5]. The European Union has implemented stringent regulations in an effort to raise public awareness about the adverse impacts (tinnitus, diarrhea, skin rashes, and even fatalities in severe cases) of KAN residues to protect human health [6]. The maximum allowable residue limit (MRL) for KAN in milk should not exceed 100 μg/kg, while the MRL is specified as 200 μg/kg in Chinese regulation [7]. A variety of techniques, including colorimetry [8,9], fluorescence [10,11], UV–vis spectroscopy [12], photoelectrochemistry [13], electrochemistry [14,15], electrochemiluminescence [16], and so on, have been proposed for the detection of KAN in recent years. Among these methods, electrochemiluminescence (ECL) has garnered attention for its low signal background, high sensitivity, great controllability, and rapid detection [17,18,19].

Normally, ECL detection is a single-signal model, which can easily lead to erroneous outcomes in complex detection environments. In comparison, employing the dual-signal output mode in ECL detection allows for effective error mitigation through self-calibration [20,21,22,23], thus improving the reliability and sensitivity of the sensor. For instance, Dai et al. [24] created europium-based organic gels (Eu-L-H MOGs) by employing europium ions as metal nodes and incorporating Luminol and 4′-(4-carboxyphenyl) -2,2′:6′,2″-terpyridine (Hcptpy) as ligands. Using dissolved oxygen and K_2_S_2_O_8_ as co-reactants, high-sensitivity detection of the I27L gene was achieved by comparing the anodic ECL signal of Luminol with the cathodic ECL signal of Hcptpy. It can be observed that dual luminophore systems generally require the introduction of dual co-reactants, thereby unavoidably complicating the detection environment and impacting the accuracy of the results. Moreover, the inherent instability of exogenous co-reactants limits the widespread application of these ECL systems. The self-enhanced ECL strategy presents a practical solution to these issues. The covalent bonding of a co-reactant with the luminophore not only substantially shortens the electron transport distance and improves ECL efficiency but also effectively mitigates the interference in the detection environment, thus improving the accuracy of the analytical results. Therefore, identifying suitable luminophores and co-reactants is crucial in the development of dual-signal self-enhanced ECL sensing systems.

In recent years, the rapid development of materials science has promoted the continuous progress of ECL, and the emergence of various luminescent materials has provided a broad space for developing dual-signal ECL sensors. Metal–organic frameworks (MOFs) have shown significant potential [25,26,27,28,29]. These crystalline structures consist of metal ions or clusters self-assembled with organic ligands, possessing a distinctive porous structure and tunability, which have advantages in the construction of sensors. In particular, zirconium-based MOFs (Zr MOFs) have garnered considerable attention due to their exceptional chemical stability and tunable porosity and have gradually occupied an important position in the construction of ECL sensors [30,31,32]. Compared with other nanomaterials, Zr MOFs exhibit superior chemical robustness under harsh environmental conditions, ensuring the long-term stability of the sensor. Additionally, the high surface area and abundant coordination sites of Zr MOFs provide ample space for co-reactant immobilization, thereby enhancing the interaction between luminophores and co-reactants. More importantly, the covalent coupling of PEI with a Zr MOF significantly shortens the electron transfer distance, which not only enhances the ECL efficiency but also reduces the interference of additional reagents in the detection environment. These features highlight the remarkable potential of Zr MOFs in constructing self-enhanced and competitive dual-signal ECL sensors. 

In this work, a Zr MOF was chosen as one of the luminophores. To enhance the ECL performance of the Zr MOF, a co-reactant (polyethyleneimine, PEI) was covalently bonded to the Zr MOF to form a Zr MOF-PEI composite, which produced an intramolecular self-enhancement effect. Luminol, as one of the most classic luminophores [33,34,35], has the advantages of low cost, good water solubility, and low excitation potential. It has a high and stable ECL emission efficiency with the presence of PEI; thus, it was selected as an additional luminophore. Based on the competition between Luminol and the Zr MOF for PEI, an appropriate amount of PEI was covalently linked to the Zr MOF and the synergistic effect of self-enhancing strategy and competition mechanism was discussed, so as to conduct accurate quantitative analysis of the target. This is crucial for the successful construction of competitive dual-signal ECL sensors.

**Scheme 1:**
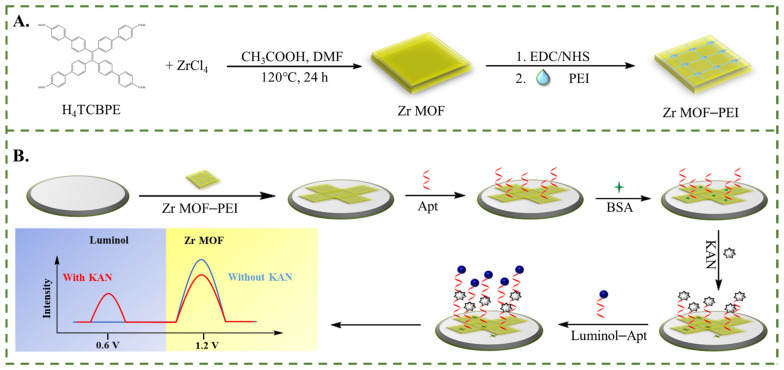
Schematic illustration for (**A**) the preparation of the Zr MOF–PEI composite and (**B**) the fabrication process of the competitive dual-signal ECL aptamer sensor.

Herein, an innovative dual-signal ECL aptamer sensor was developed for KAN detection, leveraging the competition between the Zr MOF and Luminol for the same co-reactant, as illustrated in Scheme 1. The KAN’s aptamer specifically recognized KAN and acted as a bridge between the Zr MOF-PEI and Luminol at the nanoscale to form a “sandwich” structure. The competition for PEI between the two emitters resulted in an increase of the Luminol ECL signal and a decrease of the Zr MOF ECL signal. As a result, the constructed competitive dual-signal ECL aptamer sensor exhibits excellent sensitivity and accuracy and promising potential for real sample analysis.

## 2. Experimental Section

The materials and reagents can be found in the Supplementary Material. All reagents used were of analytical grade with no further purification.

### 2.1. Preparation of Zr MOF and Zr MOF-PEI Composite

First, 60 mg of ZrCl_4_ (National Medicines Chemical Reagent Co., Ltd., Shanghai, China), 30 mg of H_4_TCBPE (Macklin Biochemical Co., Ltd., Shanghai, China), and 1 mL of CH_3_COOH (National Medicines Chemical Reagent Co., Ltd., Shanghai, China) were ultrasonically dissolved in 15 mL of DMF, followed by heating at 120 °C for 24 h. After cooling down to room temperature, the product was washed thrice with ethanol and water. Subsequently, the precipitate was centrifuged and dried under vacuum at 60 °C overnight, yielding a pale yellow powder suitable for subsequent research.

Subsequently, 1 mL of Zr MOF (1 mg/mL) was combined with 200 μL of EDC (0.4 M) (Aladdin Reagent Co., Ltd., Shanghai, China) and NHS (0.1 M) (Aladdin Reagent Co., Ltd., Shanghai, China) under magnetic stirring for 1.5 h. Next, 500 μL of PEI (1%) (Macklin Biochemical Co., Ltd., Shanghai, China) was added dropwise and stirred continuously overnight. The prepared Zr MOF-PEI was centrifuged at 11,000 rpm for 5 min and the sediment was washed thrice, redispersed in 1 mL of ultrapure water, and stored at 4 °C for later use.

### 2.2. Preparation of Luminol–Apt

The process of synthesizing Luminol–Apt can be found in the Supplementary Material (Luminol was purchased from Sigma–Aldrich, St. Louis, MO, USA).

### 2.3. Preparation of ECL Aptamer Sensors

As shown in Scheme 1B, the glassy carbon electrode (GCE) was initially polished using Al_2_O_3_ powder and sonicated in anhydrous ethanol and ultrapure water later, then eventually dried with nitrogen gas. Then, 10 μL of Zr MOF-PEI was applied onto the pre-treated GCE and air-dried at 25 °C. The obtained Zr MOF-PEI/GCE surface was dropped with 10 μL of aptamer solution and incubated at 37 °C for 2 h. To minimize non-specific adsorption, the Apt/Zr MOF-PEI/GCE was incubated with 0.5% BSA (National Medicines Chemical Reagent Co., Ltd., Shanghai, China) at 37 °C for 1 h. Following this, the Apt/Zr MOF-PEI/GCE was immersed in a KAN solution at 37 °C for 1 h. Finally, the KAN/Apt/Zr MOF-PEI/GCE was placed into a Luminol–Apt solution and incubated at 37 °C for 2 h. Then, the modified electrode was gently washed with PBS (prepared using Na_2_HPO_4_ and NaH_2_PO_4_·2H_2_O, National Medicines Chemical Reagent Co., Ltd., Shanghai, China) to obtain the ECL aptamer sensors (Luminol–Apt/KAN/Apt/Zr MOF-PEI/GCE).

## 3. Results and Discussion

### 3.1. Characterization of Zr MOF and Zr MOF-PEI

**Figure 1:**
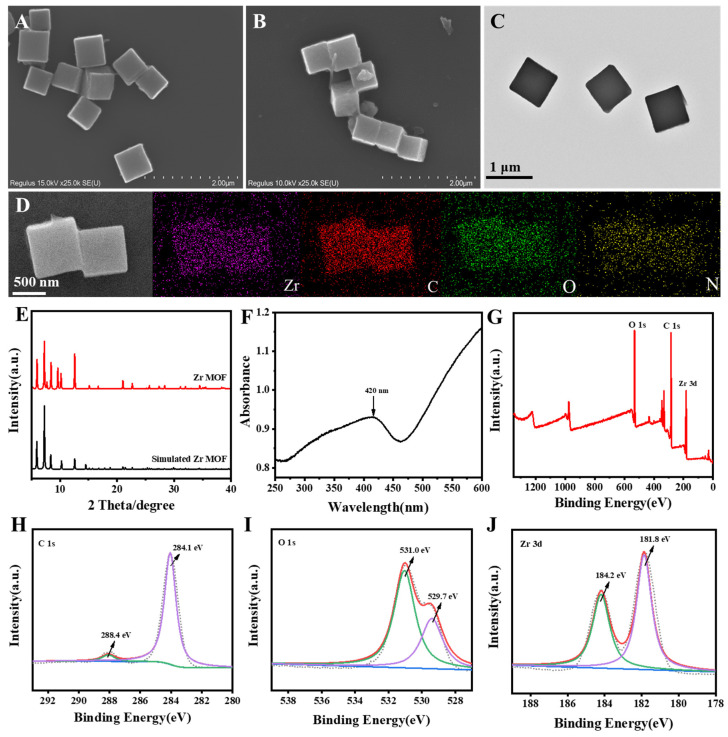
(**A**) SEM image of Zr MOF. (**B**) SEM image, (**C**) TEM image, (**D**) and element mapping images of Zr MOF-PEI. (**E**) XRD pattern and (**F**) UV–vis spectrum of Zr MOF. (**G**) XPS survey spectrum and high-resolution spectra of (**H**) O 1s, (**I**) C 1s, and (**J**) Zr 3d for Zr MOF. In (**E**), the red line represents Zr MOF and the black line represents Simulated Zr MOF.

Firstly, the morphology of the synthesized materials was examined using scanning electron microscopy (SEM). As depicted in Figure 1A, the structure of the synthesized Zr MOF exhibited a uniform cubic shape, averaging 800 nm in diameter. Due to the linkage of PEI, the previously smooth surface of the Zr MOF showed an obvious rough texture (Figure 1B). Meanwhile, the cubic morphology of Zr MOF-PEI could also be observed by transmission electron microscopy (TEM) (Figure 1C). The elemental mapping of Zr MOF-PEI (Figure 1D) depicted a uniform distribution of Zr, C, O, and N elements, verifying that the Zr MOF-PEI composite was successfully prepared. The X-ray diffraction (XRD) pattern in Figure 1E revealed that the characteristic peaks of the Zr MOF were significantly consistent with those of the simulated pattern [36]. It proved that the synthesized Zr MOF achieved the expected results. Figure 1F presented the UV–vis absorption spectrum of the Zr MOF, revealing a distinct absorption peak at 420 nm. The XPS spectra revealed the elemental composition and chemical states of the Zr-based MOF. The full spectrum, shown in Figure 1G, indicated the presence of O, C, and Zr elements. As shown in Figure 1H, the O 1s peak split into two peaks at 529.7 eV and 531.0 eV, attributed to the oxo compounds of H_4_TCBPE and the Zr-O bonds in the Zr MOF. As shown in Figure 1I, the C 1s spectrum displayed two distinct peaks at 284.1 eV and 288.4 eV, corresponding to the benzoic acid rings of the organic ligand H_4_TCBPE and the C=O bonds, respectively. In the Zr 3d spectrum (Figure 1J), the peaks fitted at binding energies of 181.8 eV and 184.2 eV were attributed to Zr 3d_5/2_ and Zr 3d_3/2_, respectively, confirming the presence of Zr (IV). These XPS results supported the formation of the MOF. Figure S1 depicts a Fourier transform infrared (FT-IR) spectral image of the Zr MOF and Zr MOF-PEI. Compared with the Zr MOF, Zr MOF-PEI exhibited a peak at 3300 cm^−1^, indicating the N-H stretching vibration, which confirmed the successful loading of PEI onto the Zr MOF. In addition, Figure S2 shows the Zeta potentials of the Zr MOF, PEI, and Zr MOF-PEI, with the changes in potential further proving the successful loading of PEI, which established the foundation for the subsequent construction of the ECL aptamer sensor. The results conclusively verified the successful preparation of the Zr MOF and Zr MOF-PEI composite.

### 3.2. Performance of the ECL Self-Enhancement and Competition Mechanism

**Figure 2:**
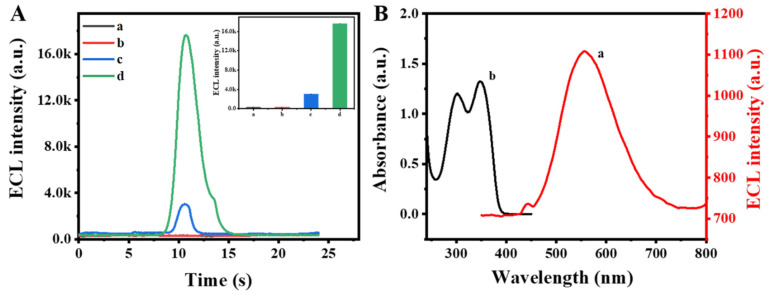
(**A**) ECL signals of (a) bare GCE, (b) Zr MOF, (c) mixture of Zr MOF and PEI, and (d) Zr MOF-PEI in PBS. (**B**) ECL spectrum of Zr MOF-PEI (a) and UV–visible absorption spectrum of Luminol (b).

The emission efficiency of ECL emitters plays a crucial role in improving sensor sensitivity. To boost the ECL signal, PEI was covalently conjugated with the Zr MOF luminophore to create a composite. Through intermolecular interaction, the electron transfer distance was shortened and energy loss minimized, leading to self-enhanced ECL. As depicted in Figure 2A, the bare GCE (curve a) and Zr MOF (curve b) exhibited negligible ECL signals in a PBS solution. However, upon the addition of PEI to the PBS solution containing the Zr MOF (curve c), a significant enhancement in the ECL signal was observed. Notably, the ECL signal of Zr MOF-PEI in PBS (curve d) was enhanced by approximately 5.8 times compared to that of the Zr MOF alone in PBS containing PEI. This remarkable enhancement, attributed to the intermolecular self-enhancement effects of Zr MOF-PEI, renders it an excellent high-intensity emitter for the development of a dual-signal competitive ECL aptamer sensor.

Furthermore, the distinct lack of spectral overlap between the ECL spectrum of the Zr MOF (curve a) and the UV–vis absorption spectrum of Luminol (curve b), as depicted in Figure 2B, was a noteworthy observation. This suggests that in the competitive dual-signal ECL aptamer sensor, the reduction of the Zr MOF signal upon the introduction of Luminol is primarily attributed to the competition for the co-reactant PEI, rather than resonance energy transfer. This key finding underscored the significance of eliminating potential interference of resonance energy transfer phenomena and establishing an accurate quantitative relationship in the competitive dual-signal ECL aptamer sensor.

**Figure 3:**
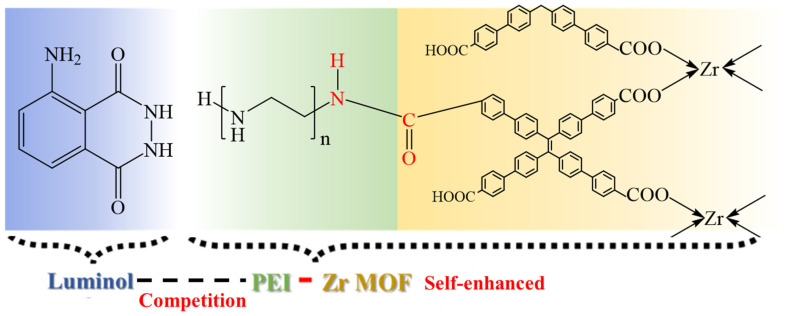
Proposed synergistic mechanism of self-enhanced ECL strategy and single co-reactant competition.

Subsequently, the synergistic mechanism of the self-enhanced ECL strategy and single co-reactant competition was deeply analyzed, as shown in Figure 3. The corresponding reaction equations were presented to explain the generation and enhancement of the ECL signals.

(1) $$Zr\;MOF-PEI-2e^{-}\to Zr\;MOF^{+}-PEI^{.+}$$

(2) $$Zr\;MOF^{+}-PEI^{.+}\to Zr\;MOF^{+}-PEI^{.}+H^{+}$$

(3) $$Zr\;MOF^{+}-PEI^{.}\to Zr\;MOF^{*}-PEI$$

(4) $$Zr\;MOF^{*}-PEI\to Zr\;MOF-PEI+{hv}_{1}$$

(5) $$Luminol-2e^{-}-2H^{+}\to Luminol^{.-}$$

(6) $$Luminol^{.-}+Zr\;MOF^{+}-PEI^{.+}\to Luminol^{*}+Products$$

(7) $$Luminol^{*}\to Luminol+{hv}_{2}$$

### 3.3. Electrochemical Characterization and ECL Behavior

**Figure 4:**
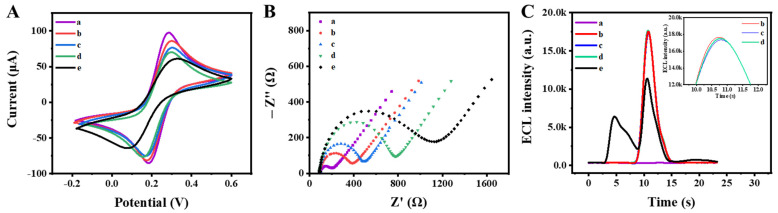
(**A**) CV and (**B**) EIS characterization of the ECL aptamer sensor in 0.1 M KCl solution containing 5.0 mM [Fe (CN)_6_] ^3−/4−^. (**C**) ECL curves of (a) bare GCE, (b) Zr MOF-PEI/GCE, (c) Apt/Zr MOF-PEI/GCE, (d) KAN/Apt/Zr MOF-PEI/GCE, and (e) Luminol–Apt/KAN/Apt/Zr MOF-PEI/GCE in PBS. The inset is a partial magnification of curves b–d.

To explore the assembly process of the ECL aptasensor, cyclic voltammetry (CV) and electrochemical impedance spectroscopy (EIS) were utilized to study the electrochemical properties. As shown in Figure 4A, the bare GCE exhibited a pair of reversible oxidation-reduction peaks (curve a). Upon modification with Zr MOF-PEI, the peak current was observed to decrease (curve b). Following the addition of the aptamer for KAN, the peak current exhibited further reduction (curve c). The subsequent binding of KAN to the aptamer significantly lowered the peak current (curve d). Finally, upon modification with the Luminol–Apt probe, the peak current experienced another decrement (curve e). The EIS spectra in Figure 4B showed a gradual increase in the electron transfer resistance (*Rct*) value at each step of the modified electrode (curves a–e), thus confirming the successful fabrication of the ECL aptamer sensor.

As shown in Figure 4C, the ECL behavior of the sensor was characterized subsequently. Compared with the bare GCE (curve a), the modification of Zr MOF−PEI produced a pronounced ECL signal (curve b). Clearly visible from the inset, the ECL signals of the Zr MOF remained stable after binding with the aptamer (curve c) and capturing KAN (curve d), which proved that the modification of the aptamer and KAN had no effect on the luminescence properties of the Zr MOF. After the introduction of the Luminol–Apt, the competition for the PEI resulted in an enhanced ECL signal of Luminol and a diminished signal of the Zr MOF (curve e). This result further validated the successful preparation of the competitive dual-signal ECL aptamer sensor.

### 3.4. Optimization of the Detection Conditions

To enhance the detection performance of the ECL aptamer sensor, a comprehensive optimization of experimental conditions was carried out. As illustrated in Figure S3, the concentration of Luminol was optimized. As the concentration of Luminol increased, the ECL signal of the Zr MOF decreased significantly and then remained stable at 12.5 mM, so it was chosen as the optimal concentration of Luminol. Additionally, the adjustment of the incubation time of the Luminol–Apt is shown in Figure S4: it took 150 min for the full binding of Luminol–Apt with KAN. When the reaction time exceeded 150 min, the intensities remained stable. Therefore, the subsequent incubation time of the Luminol–Apt was set at 150 min. The optimization of the incubation time for KAN binding to the aptamer is depicted in Figure S5. It was observed that the complete combination of KAN and aptamer occurred within 60 min, with minimal subsequent variation in the intensities. Thus, 60 min was determined to be the optimal reaction time. As shown in Figure S6, within the range of 1.0 nM to 1.0 × 10^2^ nM, the ECL intensity of Luminol increased with the increase in the concentration of the aptamer. Then, with the further increase, the ECL intensity of both the Luminol and Zr MOF decreased sharply, mainly because excessive aptamer would cover the surface of the Zr MOF-PEI, which hindered the luminescence of the Zr MOF and Luminol. Therefore, the most suitable concentration of aptamer for KAN was determined to be 1.0 × 10^2^ nM. The optimal experimental conditions obtained above were paramount in ensuring the reliability and efficiency of the aptasensor in practical applications.

### 3.5. Analytical Performance

**Figure 5:**
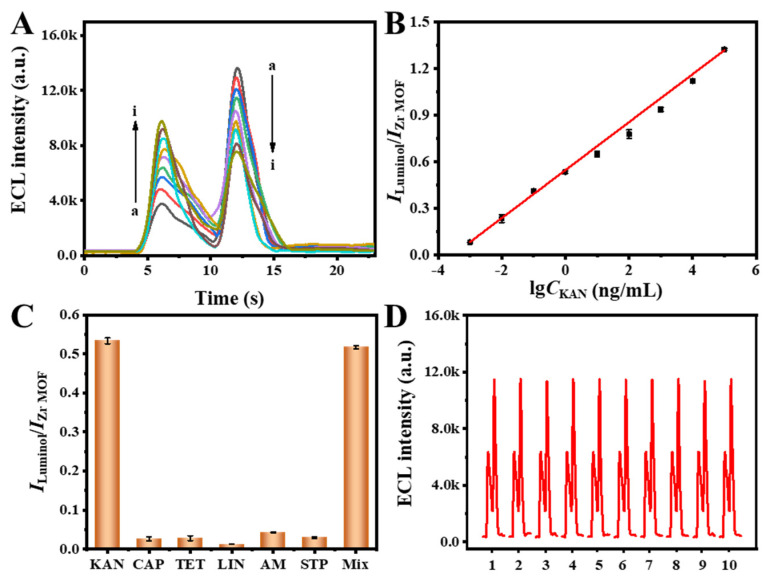
(**A**) ECL aptamer sensors’ response to various KAN concentrations: (a) 1.0 × 10^−3^ ng/mL, (b) 1.0 × 10^−2^ ng/mL, (c) 1.0 × 10^−1^ ng/mL, (d) 1.0 ng/mL, (e) 1.0 × 10^1^ ng/mL, (f) 1.0 × 10^2^ ng/mL, (g) 1.0 × 10^3^ ng/mL, (h) 1.0 × 10^4^ ng/mL, (i) 1.0 × 10^5^ ng/mL. (**B**) Calibration curve of the ECL aptamer sensor for the detection of KAN. (**C**) Selectivity (1.0 and 1.0 × 10^2^ ng/mL for KAN and other interfering analytes, respectively) and (**D**) repeatability of the ECL aptamer sensor.

Under optimized conditions, various concentrations of KAN were detected and the corresponding ECL signals were plotted (Figure 5A). As depicted in Figure 5B, the ECL intensity ratio (*I*_Luminol_/*I*_Zr MOF_) between Luminol and the Zr MOF exhibited a linear relationship with the logarithm of KAN concentration ranging from 1.0 × 10^−3^ to 1.0 × 10^5^ ng/mL. The obtained linear regression equation was *I*_Luminol_/*I*_Zr MOF_ = 0.154 lg C_KAN_ + 0.547(R^2^ = 0.998). Compared with other detection methods, the detection limit (7.86 × 10^−4^ ng/mL) of the designed sensor was lower than that reported in previous studies (Table S1), demonstrating that the dual-signal ECL aptamer sensor exhibits excellent analytical performance for KAN detection.

In addition, selectivity and repeatability are considered to be essential parameters for assessing the practicality of ECL aptamer sensors. Antibiotics such as chloramphenicol (CAP), tetracycline (TET), lincomycin (LIN), ampicillin (AM), and streptomycin (STP) were used to evaluate the selectivity of the constructed ECL aptamer sensor. The selected interferents are antibiotics typically co-existing in milk or other food samples, which may interfere with the detection of KAN in practical applications. As illustrated in Figure 5C, even when the concentrations of these interfering substances were 100-fold higher than that of KAN, they still did not significantly affect the detection of KAN, proving that the ECL aptamer sensor has satisfactory selectivity. This excellent selectivity can be attributed to the high specificity of the aptamer toward KAN. The covalent immobilization of the KAN-specific aptamer onto the Zr MOF framework and Luminol ensures strong and exclusive molecular recognition, enabling the sensor to selectively capture KAN and trigger the ECL signal predominantly in its presence while minimizing interference from other antibiotics. To further investigate the repeatability of the ECL sensor, 10 tests were performed for 1 ng/mL KAN under the same experimental conditions (Figure 5D). The repeatability was evaluated by calculating the relative standard deviation (RSD) of 10 independent measurements. The ECL signals were largely stable, the RSD of the Zr MOF was 1.27%, and the RSD of Luminol was 1.78%. Therefore, based on the high selectivity and excellent repeatability, the constructed dual-signal ECL aptamer sensor demonstrates promising potential for practical applications of detecting KAN in complex matrices.

### 3.6. Real Sample Analysis

To verify the practicability of the designed competitive dual-signal ECL aptamer sensor, KAN levels in various samples were assessed using the standard addition method to simulate the complex substrates found in food and environmental samples. The pretreatment of the real samples is shown in the Supplementary Material. As depicted in Table 1, recoveries ranged from 97.1% to 104.0% and the RSD of the ECL sensors was less than 2.9%. The results demonstrated that the ECL aptamer sensor exhibited reliability in the real sample analysis.

## 4. Conclusions

In summary, based on the competition between a Zr MOF with self-enhanced effects and Luminol for the single co-reactant PEI, we have devised a novel dual-signal ECL sensing platform for the highly sensitive detection of KAN. The exceptional performance of this platform could be attributed to several critical factors. Firstly, the covalent binding of the Zr MOF and PEI was conducive to reducing the electron transfer distance and minimizing the energy loss, thus effectively enhancing the ECL signal intensity and boosting the sensitivity. Secondly, the abundant active sites in the Zr MOF facilitated the immobilization of aptamer, leading to a higher loading of the Luminol signal probe and further enhancing sensitivity. Thirdly, the competition between the Zr MOF and Luminol for PEI introduced a dual-signal self-calibration mode, improving the precision of KAN detection. This work offers a new perspective on the synergistic mechanisms involving self-enhanced ECL and competitive ECL and opens a new approach for the rational design of dual-signal ECL sensing platforms.

## Figures and Tables

**Table 1:** Recoveries for the detection of KAN in real samples.

Sample	Spiked (ng/mL)	Found (ng/mL)	Recovery (%)	RSD (%)
Milk	0	No Found	-	-
	0.01	0.01	100	0.4
	1.00	1.00	100	1.1
	100.0	102.6	103	1.1
River water	0	No Found	-	-
	0.01	0.01	100	2.9
	1.00	1.03	103	2.2
	100.0	97.11	97.1	1.0
Honey	0	No Found	-	-
	0.01	0.01	100	1.2
	1.00	1.04	104	1.5
	100.0	98.78	98.8	0.5

## Data Availability

The experimental data are contained within this article.

## Supplementary Materials

The following supporting information can be downloaded at: https://www.mdpi.com/article/10.3390/bios15050291/s1, Figure S1: FT-IR spectra of Zr MOF and Zr MOF-PEI; Figure S2: Zeta-potential of Zr MOF-PEI complex; Figure S3: Optimization of the concentration of Luminol; Figure S4: Optimization of the incubation time of Luminol–Apt; Figure S5: Optimization of the incubation time of KAN; Figure S6: Optimization of the concentration of Apt; Table S1: Comparison of different analytical methods for KAN. References [37,38,39,40,41,42,43,44,45,46,47] are cited in the Supplementary Materials.
