# Supplementary material for: A Dual-Signal Electrochemiluminescence Sensor for Kanamycin Detection Based on a Self-Enhanced Zr MOF and Single Co-Reactant Competition Mechanism
Source: Biosensors (Basel). 2025 May 5;15(5):291. doi: 10.3390/bios15050291 (PMC12109509; doi:10.3390/bios15050291)
Supplement: Supplementary file 1 [file biosensors-15-00291-s001.zip › biosensors-3603684-supplementary.pdf]

Supplementary Material

A dual-signal electrochemiluminescence sensor for kanamycin detection based on self-enhanced Zr MOFs and single co-reactant competition mechanism

Yawen Zhu[1], Xuemei Wang[1], Zhiyong Yan[3], Feifei Zhang[1], Jianfei Xia[1], Lili Lv[1]*, Zonghua Wang[1,2]*

[1] College of Chemistry and Chemical Engineering, Qingdao Application Technology Innovation Center of Photoelectric Biosensing for Clinical Diagnosis and Treatment, Shandong Sino-Japanese Center for Collaborative Research of Carbon Nanomaterials, Instrumental Analysis Center of Qingdao University, Qingdao University, Qingdao 266071, China

[2] Characteristic Laboratory of Advanced Metal Functional Materials and Processing in Universities of Shandong, School of Mechanical and Electronic Engineering, Qingdao Binhai University, Qingdao, Shandong, 266555, China

[3] Institute of Analysis and Testing, Beijing Academy of Science and Technology (Beijing Center for Physical and Chemical Analysis), Beijing, 100089, China.

*Corresponding author.

E-mail: wangzonghua@qdu.edu.cn

[#]Yawen Zhu and Xuemei Wang contributed equally to this work.

Table of Contents

Materials and reagents……………………………………………………………………………S3

Apparatus and characterization……………………………………………………….....……S3

Synthesis of Luminol-Apt……..………………………………………………...….………..S4

Synthesis of samples…………………………………..……………………...…...…………S4

Fig. S1. FT-IR spectra of Zr MOFs and Zr MOFs-PEI.…………………………….…….…S6

Fig. S2. Zeta-potential of Zr MOFs-PEI complex…………………………………………...…S7

Fig. S3. Optimization of the concentration of Luminol……………………………..…..………S8

Fig. S4. Optimization of the incubation time of Luminol-Apt…………………………….…...S9

Fig. S5. Optimization of the incubation time of KAN………………………………………..S10

Fig. S6. Optimization of the concentration of Apt……………………………...…………S11

Table S1. Comparison of different analytical methods for KAN……………………….……S12

References………………………………………………………………………….………S13

1. Materials and reagents.

The sequence of the aptamer for KAN was 5'-COOH-TAGGGCGGCTCGCCCCCAGATGGGGGTTGAGGCTAAGCCGA-3', it was purchased from Shanghai Sangon Biological Engineering Technology & Services Co., Ltd. Acetic acid (CH_3COOH), zirconium chloride ($ZrCl_4$), ethylenediamine tetraacetic acid (EDTA), dimethyl sulfoxide (DMSO), and bovine serum albumin (BSA) were purchased from National Medicines Chemical Reagent Co., Ltd. Luminol was purchased from Sigma-Aldrich. 1,1,2,2-tetrakis(4-carboxyphenyl) ethylene (H_4TCBPE), N, N-dimethylformamide (DMF) were purchased from Macklin Chemical Reagent Co., Ltd. 1-ethyl-3-(3-dimethylaminopropyl) carbodiimide hydrochloride (EDC) and N-hydroxysuccinimide (NHS) were purchased from Aladdin Reagent (Shanghai) Co., Ltd. 0.1 M phosphate buffer saline (PBS) at different pH was prepared with Na_2HPO_4 and $NaH_2PO_4 \cdot 2H_2O$ containing 0.1 M NaCl. Ultrapure water (18.2 MΩ·cm) was used throughout the experiments. All reagents were used analytical grade with no further purification for use.

2. Apparatus and characterization

Scanning electron microscopy (SEM) images were obtained using a JSM-7401 Field Emission SEM system (JEOL, Japan). Transmission electron microscopy (TEM) measurements were carried out on the transmission electron microscope (Hitachi, Japan). UV-vis spectra were measured using a UV-3900 spectrophotometer from Hitachi (Japan). Fourier transform infrared (FT-IR) spectra were obtained using a PerkinElmer Spectrum GX spectrophotometer (PerkinElmer Co., Waltham, MA). X-

ray diffraction (XRD) patterns (λ=1.5418 Å) were collected using a D8 Advance (Bruker) X-ray diffractometer. Cyclic voltammetry (CV) was performed using a CHI660b instrument from CH Instruments, Inc. (USA). Electrochemical impedance spectroscopy (EIS) was conducted on a PARSTAT 2273 potentiostat/galvanostat (Advanced Measurement Technology Inc., USA) by applying an AC voltage amplitude of 5 mV in the frequency range from 0.1 Hz to 100 kHz in a 5 mM $K_3[Fe(CN)_6]/K_4[Fe(CN)_6]$ as the redox probe solution with 0.5 M KCl. ECL measurements were carried out on an MPI-B multifunctional electrochemical analysis system from Xi'an Remex Analytical Instrument Co., Ltd. The three-electrode system included a glassy carbon electrode (GCE), platinum wire (Pt) and a saturated Ag/AgCl electrode (sat. KCl) as the working, counter, and reference electrodes, respectively.

3. Synthesis of Luminol-Apt

The aptamer was activated with 200 μL of EDC (0.01 M) and NHS (0.02 M) at 25°C for 1 h. Subsequently, 200 μL of 12.5 mM Luminol was added, and the mixture was incubated overnight at 37°C. Finally, the excess Luminol was removed by centrifugation (12000 rpm, 30 min), and the Luminol-Apt was redispersed in PBS solution for later use.

4. Pretreament of samples

The milk sample was pretreated as follows. Initially, 400 μL of EDTA (0.5 M) was added to 2 mL of milk samples and stirred for 5 min, and then 7.6 mL of acetonitrile solution (comprising 2% formic acid and 2% DMSO) was added to the mixture to precipitate proteins. Subsequently, the mixture was centrifuged at 6000 rpm for 15 min,

and the supernatant was collected for the detection of KAN.

The honey sample was pretreated as follows. First, 2 g of honey sample was diluted by 20 mL of water, then, the mixture was centrifuged at 7500 rpm for 5 min to remove impurity, and the supernatant was taken for the detection of KAN.

The river sample was pretreated as follows. The river water sample was filtered by 0.22 μm microporous filtration membrane for the detection of KAN.

The recoveries of KAN in the real samples was detected by the standard addition method. The process involved the introduction of KAN standard solution into three pre-processed samples, resulting in samples with spiked concentrations of 0.01, 1, and 100 ng/mL, respectively.

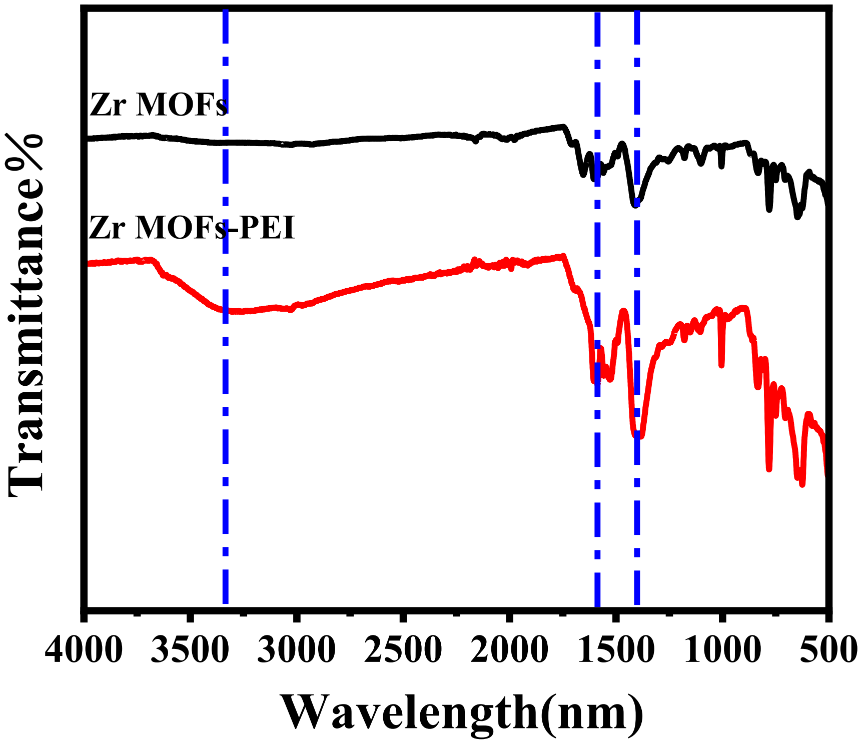

Figure S1. FT-IR spectra of Zr MOFs and Zr MOFs-PEI.

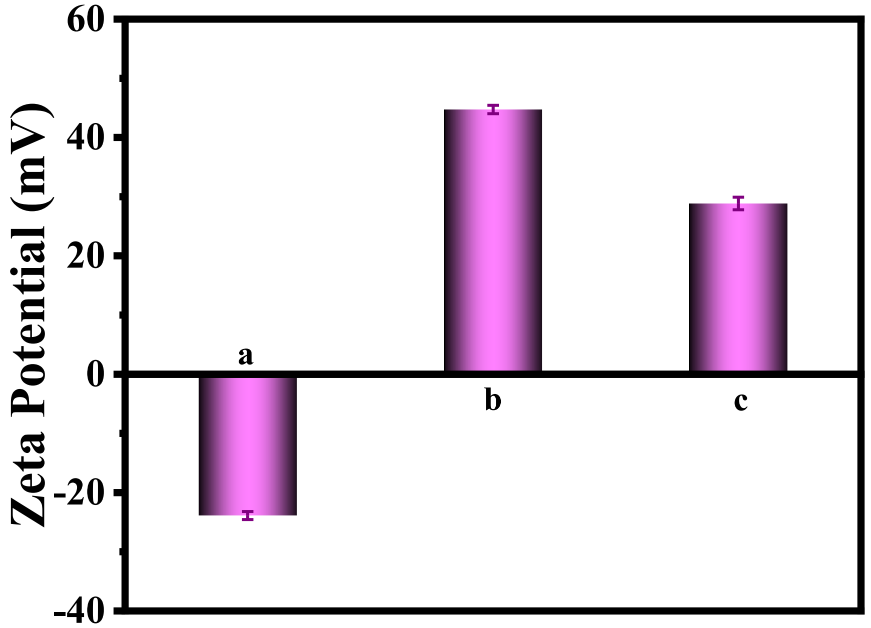

Figure S2. Zeta-potential of the Zr MOFs (a), PEI (b), and Zr MOFs-PEI composites (c).

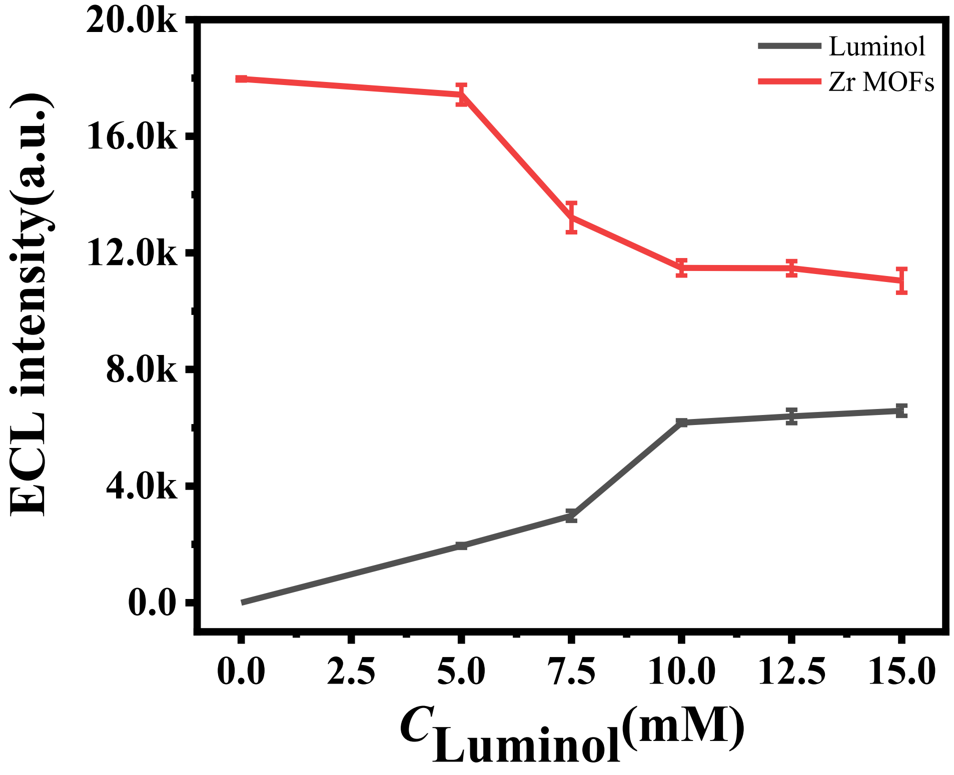

Figure S3. The ECL intensity of the biosensor with different concentration of Luminol in PBS solution. The concentration of KAN was 1.0 ng/mL. The error bars were calculated from three parallel experiments.

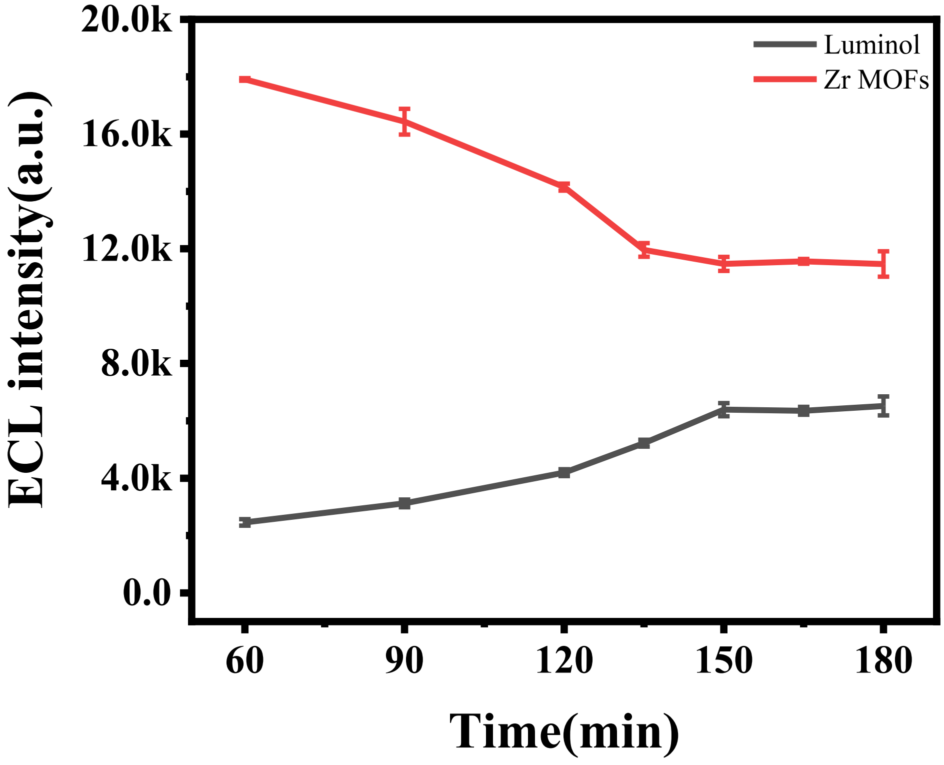

Figure S4. The ECL intensity of the biosensor with different incubation time of Luminol-Apt in PBS solution. The concentration of KAN was 1.0 ng/mL. The error bars were calculated from three parallel experiments.

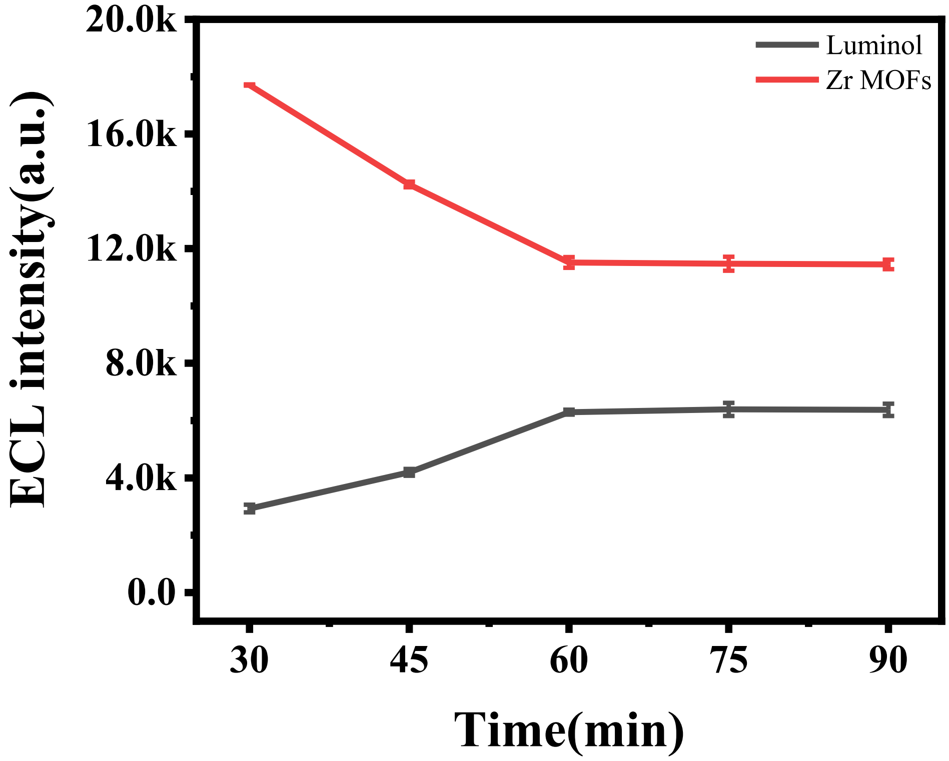

Figure S5. The ECL intensity of the biosensor with different incubation time of KAN in PBS solution. The concentration of KAN was 1.0 ng/mL. The error bars were calculated from three parallel experiments.

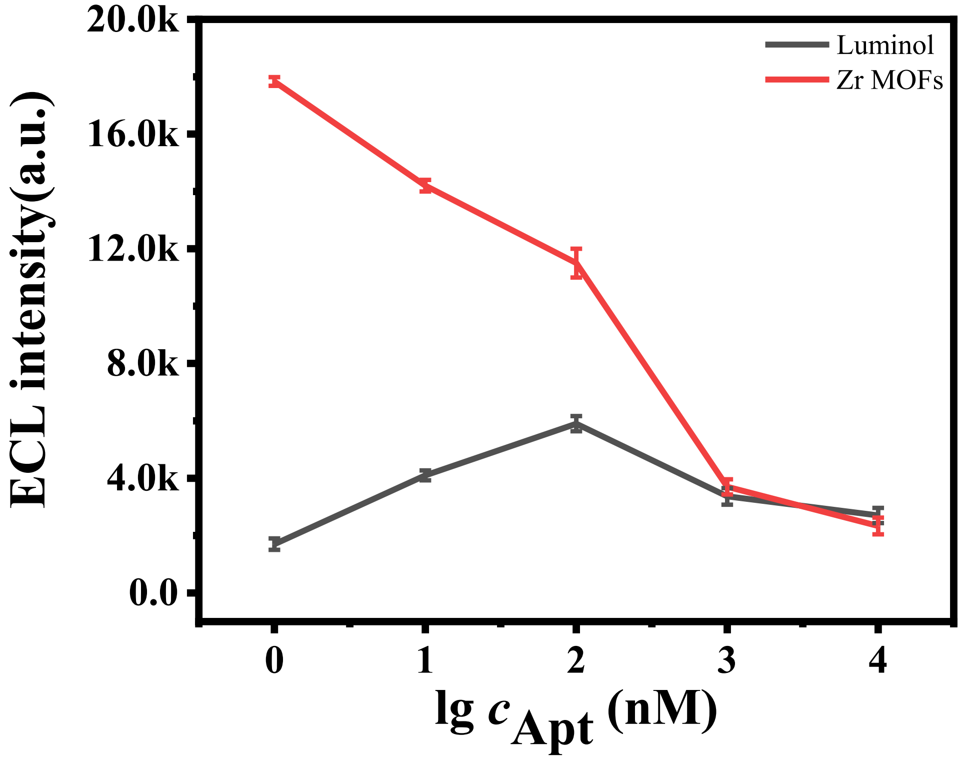

Figure S6. The ECL intensity of the biosensor with different concentration of Apt in PBS solution. The concentration of KAN was 1.0 ng/mL. The error bars were calculated from three parallel experiments.

Table S1 Comparison of different analytical methods for KAN.

Method	Linear range (ng/mL)	LOD (ng/mL)	Ref.
Photoelectrochemical	5.8×10^{-1} ~ 1.2×10^{6}	2.3×10^{-1}	[1]
Photoelectrochemical	1.2×10^{2} ~ 2.6×10^{5}	2.9×10	[2]
HPLC	1.2×10^{3} ~ 4.0×10^{4}	3.0×10^{2}	[3]
HPLC	5.0×10^{5} ~5.0×10^{7}	7.0×10^{4}	[4]
Fluorescence	2.9×10^{3} ~ 5.0×10^{5}	1.9×10	[5]
Fluorescence	2.9×10^{2} ~ 1.2×10^{4}	1.9×10^{2}	[6]
Colorimetry	5.8×10^{3} ~ 2.3×10^{6}	2.3×10^{3}	[7]
Colorimetry	2.0×10^{3} ~7.8×10^{3}	2.0×10^{3}	[8]
ECL	0.5×10^{0} ~ 1.0×10^{2}	6.0×10^{-2}	[9]
ECL	1.0×10^{-3} ~ 1.0×10^{2}	4.4×10^{-4}	[10]
ECL	5.8×10^{1} ~ 5.8×10^{5}	1.8×10	[11]
ECL	1.0×10^{-3} ~ 1.0×10^{5}	7.9×10^{-4}	**This work**

HPLC: High performance liquid chromatography

ECL: Electrochemiluminescence

References

[1] Zhang, L.; Deng, K.Q.; Zhang, H.; Li, C.X.; Wang, J.L.; Huang, H.W.; Yi, Q.F.; Zhou, H. Dual-mode photoelectrochemical/electrochemical sensor based on Z-scheme AgBr/AgI-Ag-CNTs and aptamer structure switch for the determination of kanamycin. Microchim. Acta **2022**, *417*, 189.
https://doi.org/10.1007/s00604-022-05523-y

[2] Liu, X.Q.; Liu, P.P.; Tang, Y.F.; Yang, L.W.; Li, L.L.; Qi, Z.C.; Li, D.L.; Wong, Danny K.Y. A photoelectrochemical aptasensor based on a 3D flower-like TiO_2-MoS_2-gold nanoparticle heterostructure for detection of kanamycin. Biosens. Bioelectron **2018**, *112*, 193-201.
https://doi.org/10.1016/j.bios.2018.04.041

[3] Chen, S.X.; Liang, Y.Q.; Zhou, Y.W. Analysis of kanamycin A in human plasma and in oral dosage form by derivatization with 1-naphthyl isothiocyanate and high-performance liquid chromatography. J. Sep. Sci **2006**, *29*, 607-612.
https://doi.org/10.1002/jssc.200500450

[4] Perez, J.J.; Chen, C.Y. Detection of acetyltransferase modification of kanamycin, an aminoglycoside antibiotic, in bacteria using ultrahigh-performance liquid chromatography tandem mass spectrometry. Rapid Commun. Mass Spectrom **2018**, *32*, 1549–1556.
https://doi.org/10.1002/rcm.8160

[5] Li, M.N.; Xie, Y.F.; Zhang, J.B.; Lie, L.L.; Su, X.G. Construction of a laccase mimic enzyme with fluorescence properties for kanamycin multi-mode analysis. Chem. Eng. J **2023**, *471*, 144184.

https://doi.org/10.1016/j.cej.2023.144184

[6] Ramezani, M.; Danesh, N.M.; Abnous, P.; Lavaee, K.; Taghdisi, S.M. A selective and sensitive fluorescent aptasensor for detection of kanamycin based on catalytic recycling activity of exonuclease III and gold nanoparticles. Sen. Actuators B Chem **2016**, *222*, 1-7.

https://doi.org/10.1016/j.snb.2015.08.024

[7] Xu, R.R.; Cheng, Y.Q.; Qi, X.X.; Li, X.T.; Zhang, Z.W.; Chen, L.Y.; Sun, T.; Gao, Z.H.; Zhu, M.J. Target-induced gold nanoparticles colorimetric sensing coupled with aptamer for rapid and high-sensitivity detecting kanamycin. Anal. Chem. Acta **2022**, *1230*, 340377.

https://doi.org/10.1016/j.aca.2022.340377

[8] Ha, N.R.; Jung, I.P.; Kim, S.H.; Kim, A.R.; Yoon, M.Y. Paper chip-based colorimetric sensing assay for ultra-sensitive detection of residual kanamycin. Process Biochem **2017**, *62*, 161-168.

https://doi.org/10.1016/j.procbio.2017.07.008

[9] Cheng, S.T.; Liu, H.M.; Zhang, H.; Chu, G.L.; Guo, Y.M.; Sun, X. Ultrasensitive electrochemiluminescence aptasensor for kanamycin detection based on silver nanoparticle-catalyzed chemiluminescent reaction between luminol and hydrogen peroxide. Sen. Actuators B Chem **2020**, *304*, 127367.

https://doi.org/10.1016/j.snb.2019.127367

[10] Li, H. K. ; Cai, Q. Q. ; Bai, M. H. ; Jie, G. F. Novel dual-potential color-resolved luminophore $Ru(bpy)_3^{2+}$-doped CdSe QDs for bipolar electrode electrochemiluminescence biosensing. Anal. Chem **2025**, *97*, 953−961. https://doi.org/10.1021/acs.analchem.4c05678

[11] Wang, H.Y.; Wang, Y.W.; Cai, L.; Liu, C.; Zhang, B.; Fang, G.Z.; Wang, S. Polythionine-mediated AgNWs-AuNPs aggregation conductive network: Fabrication of molecularly imprinted electrochemiluminescence sensors for selective capture of kanamycin. J. Hazard. Mater **2022**, *434*, 128882. https://doi.org/10.1016/j.jhazmat.2022.128882
